# Haptoglobin and Glutamine Synthetase May Biomark Cachexia Induced by Antiacute Myeloid Leukaemia Chemotherapy

**DOI:** 10.1002/jcsm.13849

**Published:** 2025-06-05

**Authors:** Dean G. Campelj, Cara A. Timpani, Guinevere Spiesberger, Luke E. Formosa, Joel R. Steele, Haijian Zhang, Ralf B. Schittenhelm, Lewis Leow, Craig A. Goodman, Emma Rybalka

**Affiliations:** ^1^ Institute for Health and Sport, Victoria University Melbourne Victoria Australia; ^2^ Biology of Ageing Laboratory Centre for Healthy Ageing, Centenary Institute Camperdown New South Wales Australia; ^3^ Faculty of Medicine and Health, Charles Perkins Centre University of Sydney Sydney Australia; ^4^ Inherited and Acquired Myopathies Program Australian Institute for Musculoskeletal Science St Albans Victoria Australia; ^5^ Department of Medicine—Western Health Melbourne Medical School, The University of Melbourne St Albans Victoria Australia; ^6^ Department of Biochemistry and Molecular Biology Monash Biomedicine Discovery Institute, Monash University Clayton Victoria Australia; ^7^ Monash Proteomics and Metabolomics Platform, Department of Biochemistry and Molecular Biology Monash University Clayton Victoria Australia; ^8^ Centre for Muscle Research, Department of Anatomy and Physiology The University of Melbourne Parkville Victoria Australia

**Keywords:** anticancer chemotherapy, atrophy, biomarkers, cachexia, haptoglobin, skeletal muscle wasting

## Abstract

**Background:**

Anticancer chemotherapy is an underappreciated contributor to cancer cachexia, an often‐irreversible body‐wasting condition that causes 20%–30% of cancer‐related deaths. An obstacle to predicting, monitoring and understanding the mechanisms underlying chemotherapy cachexia is that each cancer (and subtype) is assigned different chemotherapeutic compounds, typically in multiagent regimens. Here, we investigate the chemotherapy induction regimen (CIR) used in the haematological cancer, acute myeloid leukaemia (AML). We hypothesised that the AML CIR would induce cachexia, including loss of lean tissue mass and skeletal muscle atrophy.

**Methods:**

Using an unbiased proteomics approach, we interrogated the underlying molecular mechanisms. Three‐month‐old male Balb/c mice were treated with the AML CIR via intraperitoneal injections of daunorubicin (1.7 mg/kg) on Days 1–3 and cytarabine (33.2 mg/kg) administered on Days 1–7 or vehicle. Mice were assessed 24 h after the last treatment, on Day 8, or allowed to recover for 2 weeks and assessed on Day 22. A third cohort was given access to running wheels in cages. We assessed body composition and whole‐body metabolism and assessed the muscle proteome using quantitative tandem mass tag labelling LC–MS/MS analysis. Data are available via ProteomeXchange with identifier PXD063910.

**Results:**

The AML CIR‐induced acute cachexia involved a ~10% loss of body mass, ~10% loss of lean mass and ~20% reduction in skeletal muscle fibre size. Whole‐body metabolism and ambulatory activity declined. This cachexic phenotype did not recover over the 2‐week post‐CIR period (lean mass loss post‐CIR: 1 week ~7% vs. 2 weeks ~9%). In voluntarily active CIR‐treated mice, body wasting was exacerbated due to unchecked loss of fat mass (CIR sedentary: ~31% vs. CIR active: ~51%). Muscle proteome studies revealed upregulation of haptoglobin (Hp) and glutamine synthetase (Glul), which were positively correlated with body and lean mass loss. Hp was sensitive to the conditional induction, recovery and exacerbation of AML CIR‐mediated cachexia, suggestive of biomarker potential.

**Conclusions:**

The AML CIR induces an acute reduction of body, lean and fat mass underpinned by skeletal muscle atrophy, hypermetabolism and catabolism. Our data uncovered a conditionally sensitive muscle biomarker in Hp, which may be useful as a prognostic tool across other scenarios of chemotherapy‐induced myopathy and cachexia or as a target for therapeutic discovery in follow‐up studies.

## 1 Introduction

Cancer‐associated cachexia is a multifactorial wasting syndrome of body and lean tissue mass that may also include fat loss [1], and anticancer chemotherapy has gathered traction as a critical cachexia‐promoting factor [2]. Common hallmarks are skeletal muscle wasting, dysregulated metabolism and reduced food intake [1]. Patients with haematological cancers such as acute myeloid leukaemia (AML) are prone to cachexia, although the phenomenon is less studied than with solid tumours [3]. Since skeletal muscle mass is a key prognostic factor for survival in AML, preventing muscle cachexia is critical for improving survival in a disease that already has grim survival statistics [3].

Cachexia in the AML setting may, in part, be driven by the intensity of initial treatment. Universally, this comprises the ‘7 + 3’ chemotherapy induction regimen (CIR) involving 7 days of cytarabine (an antimetabolite) concomitant with anthracycline (typically daunorubicin or idarubicin) on Days 1–3 [4]. AML patients may receive multiple cycles of the CIR before complete remission is achieved. Thereafter, consolidation chemotherapy is administered, and patients are assessed for haematopoietic cell transplantation (HCT), the only current curative strategy for AML. Pre‐HCT cachexia leads to poor treatment‐related outcomes and is a growing concern for clinicians [5]. Currently, there are limited data on the impact of CIR on skeletal muscle (or on driving cachexia) and whether complete recovery from it is possible. Indeed, children with haematological cancers who receive intense chemotherapy tend not to recover their muscle mass and function, leaving survivors burdened with poor muscle health throughout life [6].

The inability to predict those likely to develop muscle cachexia and those who manifest early symptoms hampers the clinical treatment of cancer and other chronic diseases in which cachexia is devastating. In this regard, quantitative proteomics enables the detection of protein biomarkers that become disproportionate relative to the muscle‐specific proteome in disease states. These proteins have the potential to not only indicate the likelihood and scope of cachexia impact but to also predict the likelihood that cachexia could be life‐threatening and assess the effectiveness of potential therapeutics. In this study, we sought to identify putative protein biomarkers of cachexia via quantitative tandem mass tag (TMT)–labelling proteomics. We hypothesised, in line with data for the procachexia anthracycline analogue, doxorubicin [7], that the AML CIR would drive cachexia, including skeletal muscle wasting.

## 2 Methods

### 2.1 Experimental Protocols and Treatments

#### 2.1.1 Animals

Three‐month‐old (sexually mature) male Balb/c mice were acquired from the Animal Resource Centre (now Ozgene, Western Australia). Sex was controlled since AML is more prevalent in males. Mice were housed on a 12‐h light/dark cycle with ad libitum access to standard rodent chow and water.

#### 2.1.2 Chemotherapy

Mice were randomly allocated to treatment groups: intraperitoneal injections of daunorubicin (1.7 mg/kg) on Days 1–3 and cytarabine (33.2 mg/kg) on Days 1–7, or delivery vehicle (VEH; 0.9% saline) daily (total *n* = 20, 10/group). Daunorubicin dose was the maximum tolerable dose derived from our pilot studies based on dose escalation from a published starting dose [8]. Cytarabine dose was equivalent to clinical AML treatment [9] adjusted for mice based on FDA guidelines [10]. Mice were individually housed in metabolic cages to assess ambulatory and metabolic activity during CIR treatment. The experimental endpoint was 24 h after the final chemotherapy treatment (i.e., Day 8).

#### 2.1.3 Recovery From Chemotherapy

Communally housed mice (*n* = 48, *n* = 8/group, *n* = 4–5/cage) received VEH or CIR treatment as stated above were assessed for recovery over time at 24 h (Day 8), 1 week (Day 15) and 2 weeks (Day 22) after the final CIR treatment. There were no interventions during recovery.

#### 2.1.4 Biomarker Lability Using Exercise

VEH and CIR‐treated mice were randomly allocated to individual metabolic cages containing a running wheel (total *n* = 10/group). Active (ACT) mice had ad libitum access to the running wheel throughout treatment, whereas the running wheel was locked for sedentary (SED) mice.

### 2.2 Pre‐ and Postmortem Animal Analyses

#### 2.2.1 Indirect Calorimetry and Activity Monitoring

Promethion metabolic cages fitted with laser tracking capacity and running wheels (Sable Systems, United States) were used to assess cage‐ and wheel‐based physical activity and whole‐body metabolism in real time and to apply exercise. Mice acclimatised for 3 days, and data collection occurred on Days 4–10 across the 7‐day treatment period [11].

#### 2.2.2 Body Composition Analysis

Echo magnetic resonance imaging (echoMRI; EMR‐150, Echo Medical Systems, United States) was used to assess body composition [11]. Live mice were scanned on Day 1 (pretreatment), Day 8 (post‐treatment), and, for recovery experiments, additionally on Days 15 and 22 of the experiment.

#### 2.2.3 Tissue Collection

Twenty‐four hours after the last CIR treatment on Day 7 and live analyses on Day 8, mice were deeply anaesthetised with isoflurane (5% induction and 2%–3% maintenance). Muscles and organs were surgically removed, weighed and snap frozen. Tibialis anterior (TA) muscles were prepared for histology.

#### 2.2.4 Histological Analyses

TA muscles were cryopreserved in optimal cutting temperature compound (Sakura Finetek) using liquid nitrogen‐cooled isopentane and cryosectioned at 8 μm (−18°C, Leica CM1950). Mounted sections were stained with haematoxylin and eosin (H&E) or picrosirius red to assess fibre size and myopathy and fibrosis, respectively. Slides were imaged on a Zeiss Axio Imager Z2 microscope (GmbH, Germany) at 20× magnification. ImageJ software (NIH, United States) was used for data acquisition, as performed previously [11].

#### 2.2.5 TMT–Labelled Proteomics and Bioinformatics

TMT labelling proteomics on quadriceps samples (*n* = 6/group) were performed as previously described [12] as per Monash Proteomics and Metabolomics Platform data‐dependent methodology [13]. Bioinformatics, Reactome (v83)‐based pathways enrichment and deep pathways probing were performed as previously described [12]. Our analysis approach enables the detection of alterations in pathways that could remain unobserved when scoping only differentially expressed proteins, because individual proteins might not provide comprehensive insights into how a specific pathway is changing when assessed through global, untargeted proteomic‐level statistical comparisons. The FDR of < 0.05 is reported. The mass spectrometry proteomics data have been deposited to the ProteomeXchange Consortium via the PRIDE [14] partner repository with the dataset identifier PXD063910.

### 2.3 Statistics

Data are presented as mean ± standard error of the mean and were analysed with GraphPad Prism (v8, CA, United States; *α* = 0.05). CIR versus VEH comparisons were analysed by *t*‐test or two‐way ANOVA (with repeated measures) as necessary. For CIR versus VEH recovery time course, data were analysed by repeated measures two‐way ANOVA or mixed‐effects analysis. For physical activity effects, two‐way ANOVA (with repeated measures where necessary) was used. Tukey's or Bonferroni's post hoc testing was applied to detect between‐group effects. Proteomics statistics are stated above. Linear regression correlations were performed on pooled sample sets to determine associations between biomarker proteins and anthropometric and secondary biomarker parameters. In the correlates, data points and regression lines marked grey denote VEH and CIR groups under both 24 h post‐treatment and 2 weeks postrecovery conditions, whereas red data points and regression lines denote 24 h post VEH and CIR treatment only with recovery data points removed.

## 3 Results

### 3.1 AML CIR Induces Acute Cachexia in Mice

**FIGURE 1:**
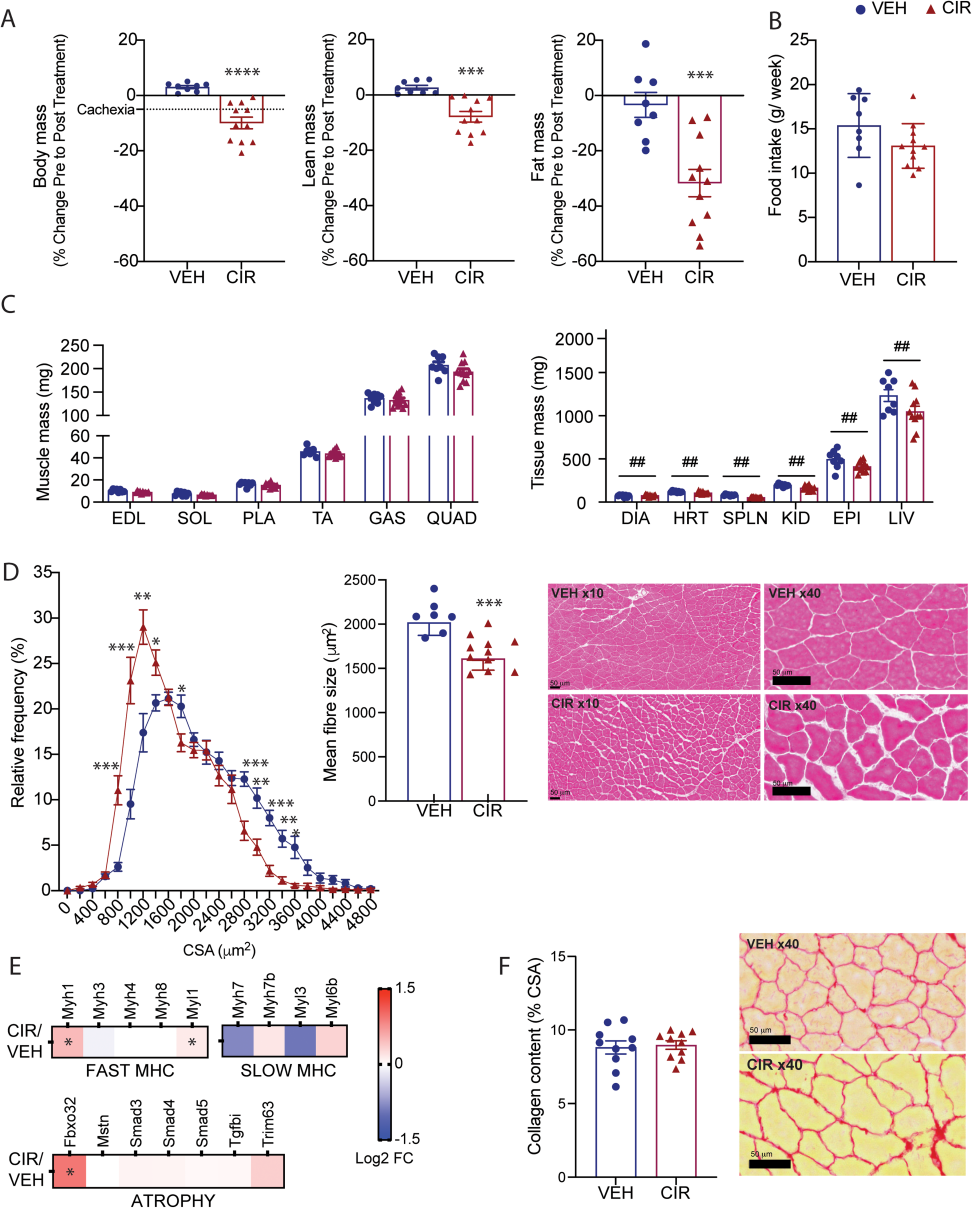
Clinically compatible cachexia profile of mice treated with antiacute myeloid leukaemia (AML) chemotherapy. Pre‐ to post‐treatment changes in (A) body mass and composition of lean and fat mass. (B) Average daily food intake over the treatment protocol. (C) Effect of treatment on endpoint muscle and tissue mass and on (D) tibialis anterior (TA) fibre size distribution, mean fibre size and representative H&E‐stained images. (E) Protein expression of fast and slow myosin heavy chain (MHC) isoforms and traditional atrophy signalling markers, atrogin‐1 (Fbxo32), TGFβ‐myostatin‐Smad axis and Murf‐1 (Trim63). (F) Collagen content and representative picrosirius stained images of TA. *p < 0.05, **p < 0.01, ***p < 0.001 and ****p < 0.0001 CIR different from VEH. #*p* < 0.05 and ##*p* < 0.01 main CIR effect. Scale bar for H&E images = 50 μm. VEH, vehicle; CIR, chemotherapy induction regimen; EDL, extensor digitorum longus; SOL, soleus; PLA, plantaris; TA, tibialis anterior; GAS, gastrocnemius; QUAD, quadriceps; DIA, diaphragm; HRT, heart; SPLN, spleen; KID, kidney; EPI, epididymal fat; LIV, liver; CSA, cross sectional area; MHC, myosin heavy chain.

AML CIR caused body mass decrements of 10%. Lean mass reduced by ~10% and fat mass by ~30% between pre‐ and post‐treatment assessments (Figure 1A). Body wasting was not entirely explained by reduced caloric intake since mean food intake in CIR mice was not statistically significant (Figure 1B). The mass of the extensor digitorum longus (EDL), soleus, plantaris, TA, gastrocnemius and quadriceps muscles was also reduced (Figure 1C). However, when corrected for body mass, there was no effect of CIR (Table S1), highlighting that muscle wasting is proportionate to the overall rate of body wasting. CIR also reduced the mass of all organs assessed (Figure 1C). In summary, our data show that AML CIR induces clinically defined cachexia in mice.

Histological fibre sizing of TA muscles revealed CIR induced muscle atrophy (Figure 1D), with more small fibres (800–1400 mm^2^) and fewer large fibres (2800–3600 mm^2^; Figure 1D). Overall, CIR reduced the mean fibre CSA by ~20% (Figure 1D). Through probing our TMT‐labelled proteomics data set (quadriceps muscle), we detected significant (based on FDR from pathways enrichment) upregulation of fast‐twitch fibre‐specific MyH isoforms, Myh1 and Myl, and of the atrophy regulator, Atrogin‐1, indicating that fast‐type fibre transformations may attempt to compensate for global fibre atrophy (Figure 1E). There was no effect of CIR on E3 ubiquitin‐protein ligase, muscle RING‐finger protein‐1 (MuRF‐1/Trim63) or components of the inducible transforming growth factor β (Tgfbi)/myostatin (Mstn)/suppressor of mothers against decapentaplegic (Smad) axis (Figure 1E). There were no signs of active necrosis or regeneration that would indicate CIR‐induced muscle damage (Figure 1D) or of fibrotic myopathy (Figure 1F).

### 3.2 AML CIR reduces Physical Activity and Systemic Energy Expenditure

**FIGURE 2:**
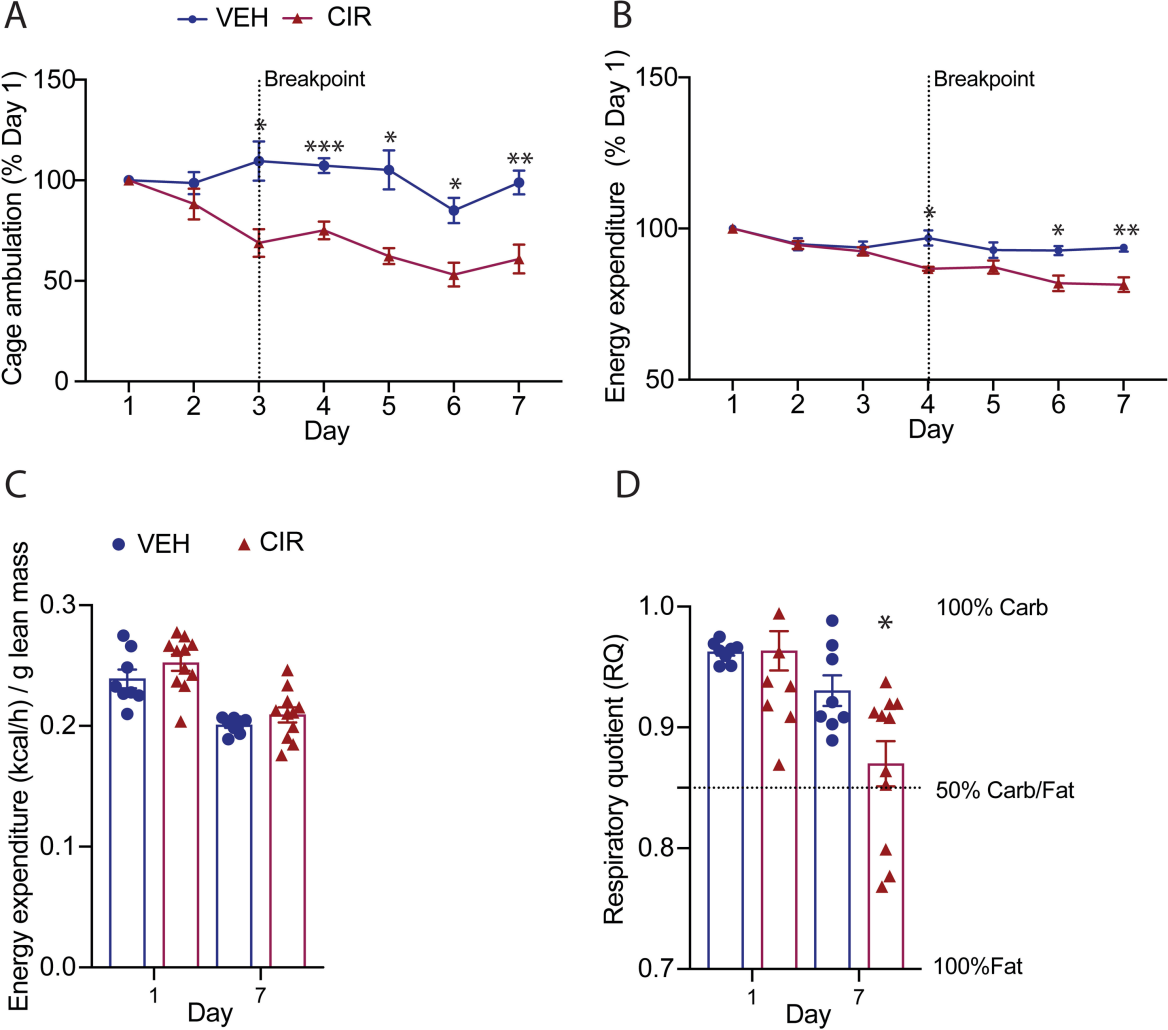
Physical activity and whole‐body metabolism are reduced in mice treated with acute myeloid leukaemia (AML) chemotherapy induction regimen (CIR). Mice were assessed continuously for (A) cage ambulatory distance and (B) energy expenditure, which is expressed relative to Day 1 levels. (C) Start‐ and end‐point energy expenditure (kilocalorie) expressed relative to lean tissue mass, and (D) the respiratory quotient (VCO_2_/VO_2_) are shown. Data are mean ± SEM. *p < 0.005, **p < 0.01 and ***p < 0.001 CIR versus VEH.

Cage‐based activity tracking revealed CIR mice reduced their physical activity levels from Day 3 of treatment and maintained significantly lower levels for the duration of the treatment period compared to VEH mice (Figure 2A). Interestingly, energy expenditure only decreased from Day 4 of treatment, indicating a 24 h window where energy expenditure is increased relative to physical activity levels (Figure 2B). However, lean tissue mass‐corrected energy expenditure was not different between VEH and CIR‐treated mice (Figure 2C), indicating energy expenditure is relative to lean tissue mass and physical activity may reduce to conserve both. Since both fat and lean mass catabolism were implicated in CIR‐induced body wasting (Figure 1), we calculated the respiratory quotient (RQ; VCO_2_/O_2_), a marker of preferential substrate utilisation and shifts thereof. The RQ revealed a significant shift in fat relative to carbohydrate metabolism in CIR mice at Day 7 (Figure 2D).

### 3.3 Haptoglobin (Hp) and Glutamine Synthetase (Glul) Are Responsive to AML CIR‐Induced Muscle cachexia

**FIGURE 3:**
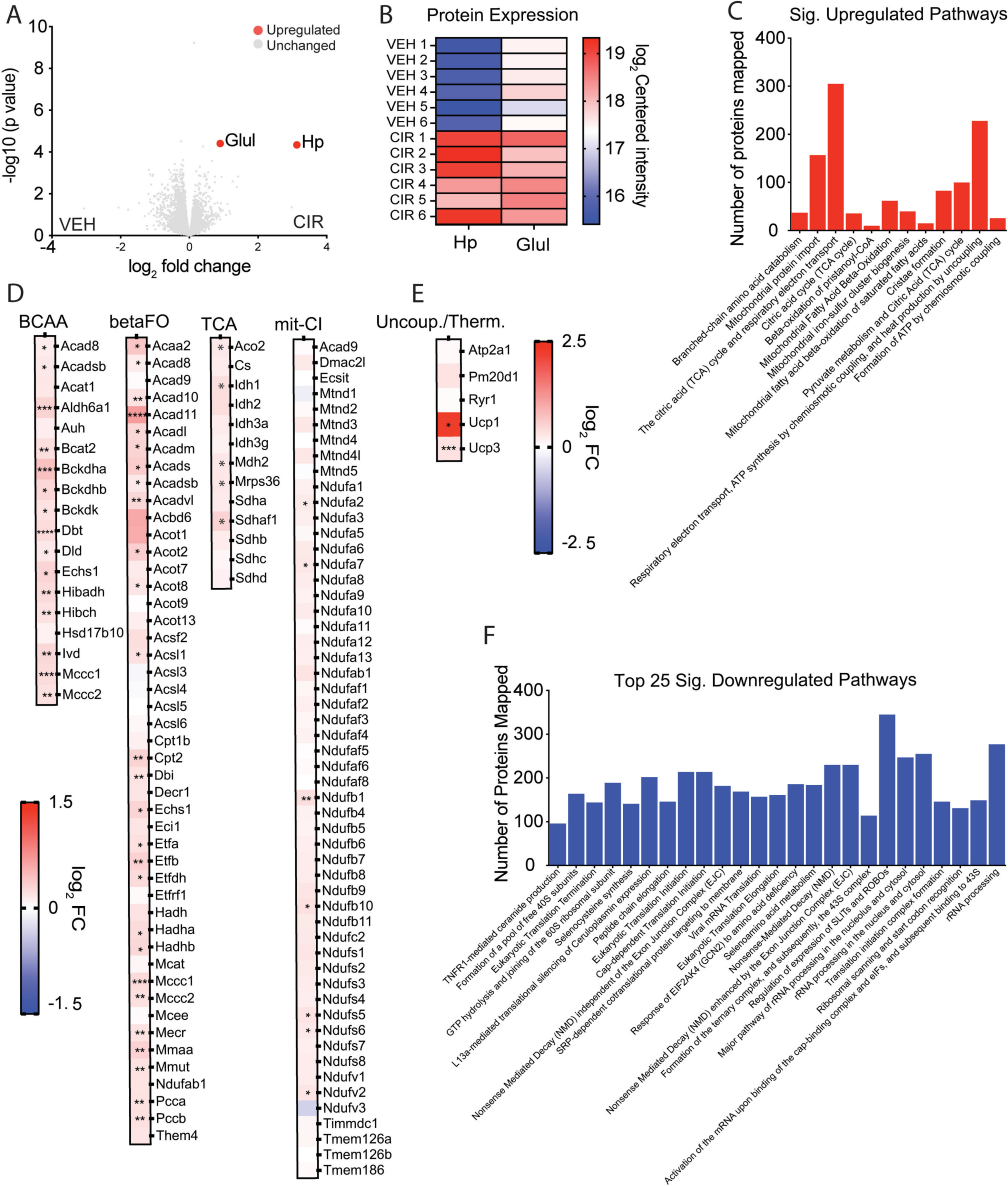
Muscle proteome response to acute myeloid leukaemia (AML) chemotherapy induction regimen (CIR) reveals haptoglobin (Hp) and glutamine synthetase (Glul) as potential biomarkers. (A) Volcano plot showing upregulation of Hp and Glul based on 0.75 log_2_‐fold change and adjusted *p* < 0.05 cut‐off. (B) Heatmap of individual mouse muscle expression of Hp and Glul. (C) Significantly upregulated Reactome pathways. (D) Protein expression profile of branched chain amino acid (BCAA) catabolism and β‐fat oxidation, mitochondrial tricarboxylic acid (TCA) cycle and mitochondrial Complex I (mit‐CI) metabolism Reactome pathways. (E) Uncoupling‐related protein expression. (F) Top 25 downregulated Reactome pathways in response to AML CIR treatment. *p < 0.05, **p < 0.01, ***p < 0.001, and ****p < 0.0001 CIR versus vehicle VEH based on *p* < 0.05.

TMT‐labelling proteomics enabled us to probe the molecular response underlying CIR‐induced muscle wasting and to identify potential muscle‐specific biomarkers. Of the 4716 proteins detected, only Hp and Glul were differentially expressed in response to AML CIR treatment according to our cut‐off criteria (log_2_ 0.75‐fold change, adjusted *p* < 0.05), and both were upregulated (Figure 3A,B). Five additional proteins were different based on adjusted *p* (< 0.05) only: Acad11 (acyl‐coA dehydrogenase family member 11), Ca14 (carbonic anhydrase 14), Cd36 (platelet glycoprotein 4) and Plin4 (perilipin 4) were upregulated, while Dgkz (diacylglycerol kinase zeta) was downregulated. Pathway enrichment identified 12 significantly upregulated pathways in response to AML CIR (based on FDR; Figure 3C) and all involved mitochondrial metabolism of amino acids, glycolytic and fatty acid substrates. The most significantly dysregulated pathway was branched chain amino acid (BCAA) catabolism, consistent with the loss of muscle mass observed. We deeply probed the BCAA, β‐oxidation and mitochondrial tricarboxylic acid (TCA) cycle pathways to decipher mechanisms (Figure 3D). Of the 18 proteins within the BCAA catabolism pathway, our proteomics detected 14 (78%) upregulated by CIR. The most significantly upregulated protein was branched chain keto acid dehydrogenase E1 subunit a (Bckdha), an inner mitochondrial protein involved in the catabolism of leucine, isoleucine and valine. In fact, most of the 14 upregulated BCAA proteins were linked to the catabolism of valine or leucine. There were 47 oxidation‐related proteins captured in our proteome, and 27 were upregulated (57%) by AML CIR treatment. Notably, almost all family members of acyl‐coA dehydrogenase (Acad), an enzyme involved in the metabolism of acyl‐coA variants, were upregulated (as was Acad6A1 in the BCAA pathways). Acad11, a recently characterised 4‐hydroxy acid (4‐HA) Acad that localises to peroxisomes and plays an important role in facilitating longer‐chain 4‐HA catabolism [15], was the most significantly upregulated protein within the β‐oxidation pathway. Several protein subunits of the mitochondrial TCA cycle were upregulated (aconitase (Aco2), isocitrate dehydrogenase (Idh1) and malate dehydrogenase (Mdh2)), although notably the TCA pacesetter and mitobiogenesis biomarker, citrate synthase (Cs), was not. Our data suggest a remodelling of mitochondria and peroxisomal metabolism to drive BCAA (especially valine and iso‐/leucine) and fat oxidation. Since anthracyclines are metabolised by mitochondrial Complex I (mit‐CI) [16, 17], we also probed this pathway. Specific components of NADH‐ubiquinone oxidoreductase (Nduf) were upregulated, including core subunit V2 (Ndufv2), which is known to protect against doxorubicin‐induced mitochondrial dysfunction‐driven cardiomyopathy [18]. Uncoupling‐related proteins were also probed as a potential mechanism of hypermetabolism (Figure 3E). Uncoupling proteins 1 and 3 (Ucp1, 3) were both upregulated by CIR and Ucp1 (brown fat specific) more so than Ucp3 (muscle specific) indicating thermogenesis as a mechanism. Recently, sarcolipin (Sln)–induced thermogenesis involving activation of the ryanodine receptor (Ryr1) and sarco (endo)plasmic reticular calcium ATPase 1 (Atp2a1) was identified as a muscle specific mechanism for nonshivering thermogenesis following exhaustion of brown fat activity [19]. These proteins were unaffected by CIR.

One hundred and thirty‐five Reactome pathways were downregulated in CIR versus VEH muscle (Table S2), and the Top 25 pathways are presented in Figure 3F. Many of the downregulated pathways were associated with ribosomal protein translation and synthesis, and the topmost downregulated pathway (based on FDR from pathways enrichment) was inflammation‐dependent (via tumour necrosis factor receptor 1 (Tnfr1)) ceramide production. In fact, several pathways concerning inflammation regulator nuclear factor kappa B (Nf‐κb) activity were reduced. Of note, the macroautophagy pathway, which when conditionally up‐ or downregulated can induce muscle atrophy [20], was significantly downregulated. Collectively, our data indicate that muscle protein synthesis pathways are suppressed, and catabolism is increased to provide substrates for mitochondrial metabolism.

### 3.4 Fat, but Not Lean Mass, Recovers in the 2 Weeks Following AML CIR

**FIGURE 4:**
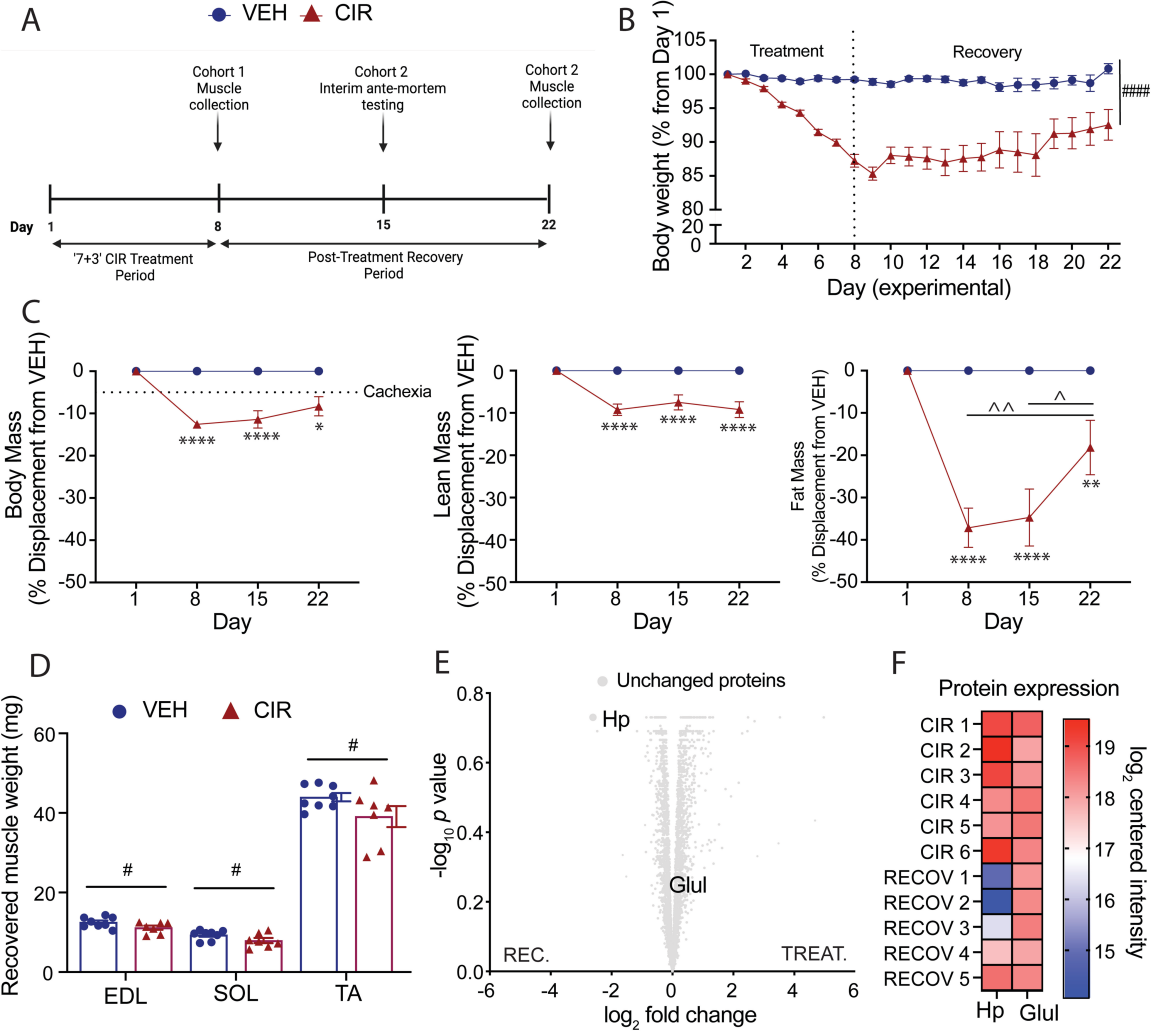
Recovery profile following of acute myeloid leukaemia (AML) chemotherapy induction regimen (CIR). (A) Experimental timeline. (B) Body mass over the AML CIR and recovery time course. (C) Body, lean and fat mass displacement relative to vehicle (VEH) over the recovery time course. (D) Endpoint muscle mass of the extensor digitorum longus (EDL), soleus (SOL) and tibialis anterior (TA). (E) Volcano plot of unresponsive quadriceps muscle proteome to CIR cessation after 2 weeks based on log_2_‐fold change and adjusted *p* < 0.05 cut‐off. (F) Haptoglobin (Hp) and glutamine synthetase (Glul) expression at the treatment (CIR) and recovery (CIR RECOV) endpoints for individual animals. *p < 0.05, **p < 0.01, and ****p < 0.0001 CIR RECOV relative to age‐matched VEH; #*p* < 0.05 and ####*p* < 0.0001 main group effect; ^*p* < 0.05 and ^^*p* < 0.01 within group time effect.

To determine whether body, lean and fat mass could recover after chemotherapy treatment, we next treated a cohort of mice with AML CIR, then allowed 2 weeks of untreated recovery during which body mass and composition were assessed daily and weekly, respectively (Figure 4A). Body mass was lowest at Day 9 (2 days into recovery; ~80% of starting body mass) and began to recover from Day 10 (Figure 4B). However, by the end of the 2‐week recovery period, body mass had not fully recovered, remaining at 90%–95% of the starting body mass. Body mass displacement from the VEH group was greatest on Day 8 and least on Day 22 of recovery; however, there was no significant difference between assessment time points in the CIR group (Figure 4C). Body mass did not recover sufficiently to renounce the clinical definition of cachexia. Lean mass was more resistant to recovery than fat mass. While fat mass displacement from VEH was still statistically different at Day 22 of recovery, it did partially recover between Days 8 and 22 by ~50%, whereas lean mass displacement did not shift (Figure 4C). There was no recovery of EDL, soleus or TA muscle mass observed (Figure 4D). The muscle proteome was also resistant. There was no significant change in muscle Hp or Glul expression at Day 22 based on log‐fold change and adjusted *p* value post recovery (Figure 4E). However, there were shifts in quadriceps Hp expression for 3/5 animals, indicating Hp is responsive to withdrawal of CIR and may biomark early changes in molecular signalling within muscle (Figure 4E). In contrast, Glul was nonresponsive to CIR withdrawal (Figure 4E).

### 3.5 Voluntary Exercise During AML CIR Treatment Worsens Cachexia and Further Upregulates Hp

**FIGURE 5:**
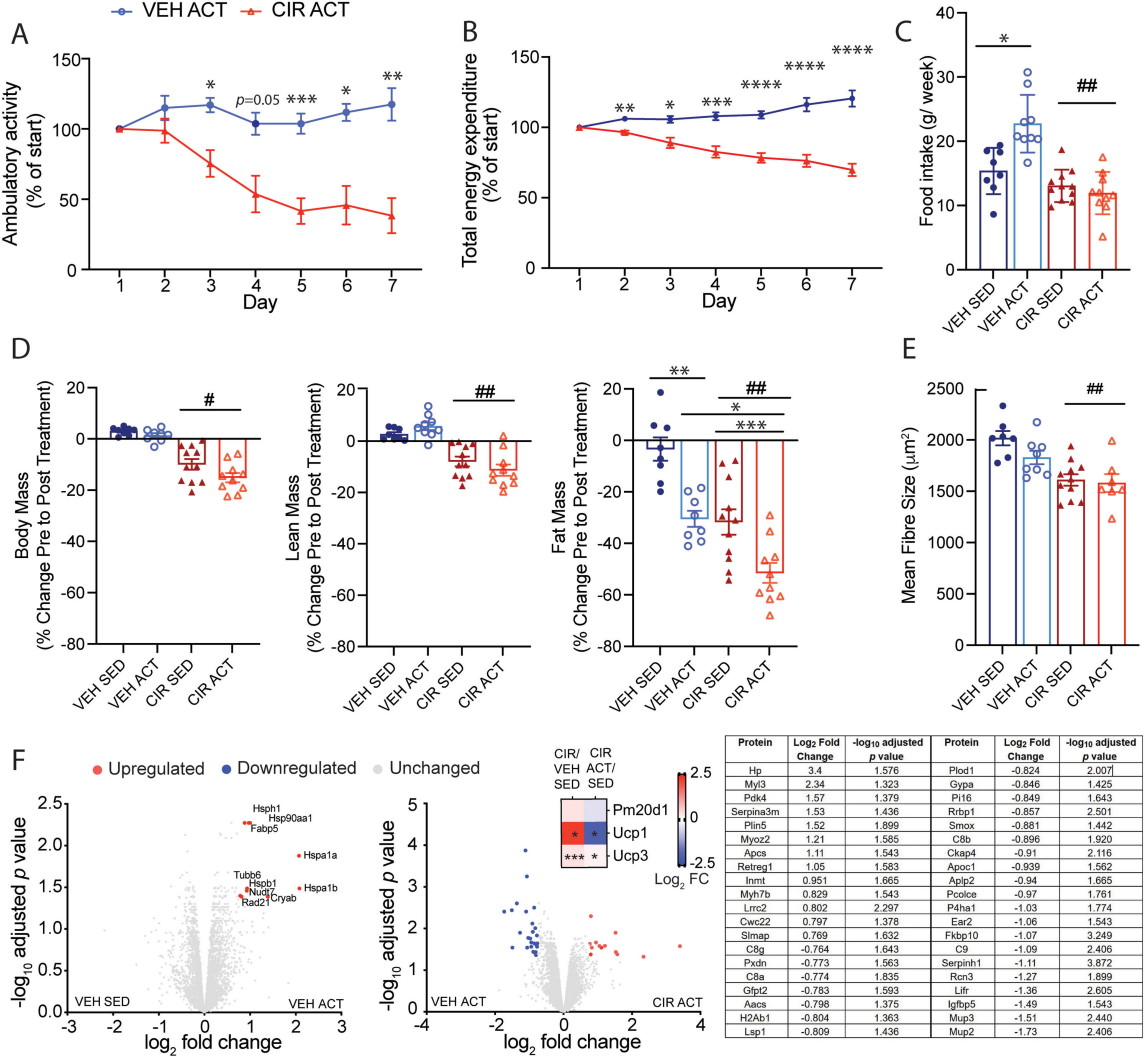
Worsening of cachexic fat mass loss by voluntary exercise during acute myeloid leukaemia (AML) chemotherapy induction regimen (CIR) treatment further upregulates muscle haptoglobin (Hp) expression. (A) Voluntary physical activity (wheel running) reduces over the AML CIR treatment period alongside (B) reduced total energy expenditure. (C) Daily food intake and (D) change in body, lean and fat mass over the treatment period. (E) Mean tibialis anterior fibre size and (F) quadriceps proteome response to voluntary exercise in vehicle (VEH) and CIR‐treated mice based on log_2_‐fold change and adjusted *p* < 0.05, including reduction of uncoupling protein (Ucp) expression based on FDR < 0.05 from pathway analysis. *p < 0.05, **p < 0.01 and ***p < 0.001 active (ACT) versus sedentary (SED) and CIR versus VEH ACT; #*p* < 0.05 and ##*p* < 0.01 main treatment effect.

We used ad libitum voluntary running activity over the 7‐day AML CIR to test (1) potential consequences during CIR and (2) the lability of Hp and Glul as cachexia biomarkers. On average, VEH ACT mice covered ~100× more distance on the running wheel compared to ground metres covered by VEH SED mice, while CIR ACT mice covered ~60× more than CIR SED mice (data not shown). From Day 3 of treatment, CIR ACT mice became steadily less active (ground and wheel metres) with endpoint physical activity < 50% of starting activity levels relative to VEH ACT (Figure 5A). Energy expenditure reduced proportionate to ambulatory distance, although notably, a day earlier than physical activity decline (Figure 5B). In healthy VEH mice, food intake increased to match the additional energy expenditure from increased physical activity (Figure 5C) sufficient to resist changes in body mass (Figure 5D). In contrast, CIR ACT mice did not increase food consumption to meet energy expenditure (Figure 5B,C). While incremental changes in body and lean mass were too small to be statistically different, CIR ACT mice trended to lose body and lean tissue mass (significant CIR group effects), whereas VEH ACT mice tended to reduce body mass but increase lean mass (Figure 5D). The most significant effect of increased physical activity was on fat mass. VEH ACT mice lost ~30% of their pre‐ACT fat mass, whereas CIR ACT mice lost ~50% for less activity. At the muscle level, the fast‐twitch glycolytic Type 2 EDL muscle of CIR ACT mice increased in size—the predominantly slow‐twitch oxidative Type 1 soleus did not change (Table 1). In contrast, in WT ACT mice, the EDL mass did not shift, but the soleus mass increased (Table 1). There was no effect of exercise on mass (Table 1) or mean fibre size (Figure 5E) of the mixed fibre type TA muscle in either VEH or CIR‐treated mice.

**TABLE 1:** Response of muscle mass to voluntary exercise during acute myeloid leukaemia (AML) chemotherapy induction regimen (CIR). Data are mean delta % change active (ACT) relative to sedentary (SED) ± SEM. EDL, extensor digitorum longus; SOL, soleus; TA, tibialis anterior. *p < 0.05 and ****p < 0.0001.

	Δ % change VEH ACT vs. SED	Δ % change CIR ACT vs. SED
EDL	5.75 ± 3.03	40.41 ± 6.54****
SOL	14.99 ± 3.12	0.44 ± 5.79*
TA	1.93 ± 2.74	−2.26 ± 1.37

Physical activity induced a unique proteomic signature (10/4766 differentially regulated proteins) in VEH mouse quadriceps involving upregulation of heat shock, DNA repair, muscle regeneration and fatty acid metabolism proteins (Figure 5F). Relative to VEH ACT, the CIR ACT proteome was considerably more impacted, with 40 differentially regulated proteins detected (13 upregulated and 27 downregulated). The most upregulated protein was Hp, which was 6.8‐fold higher than VEH following exercise exposure (i.e., CIR ACT vs. VEH ACT) compared with 5.8‐fold higher in SED mice (i.e., CIR SED vs. VEH SED). Other upregulated proteins were associated with slow fibre isoform, extracellular matrix remodelling, muscle regeneration and energy metabolism. Downregulated proteins were associated with immunogenicity/inflammation, endoplasmic reticulum proliferation, collagen biosynthesis, and polyamine‐fatty acid and cholesterol biosynthesis indicative of a whole cell wasting phenotype. Ucp1 expression was normalised, and Ucp3 expression was downregulated by exercise. Muscle Glul expression was not different in the AML CIR ACT relative to the VEH ACT group.

### 3.6 Interrogating Hp and Glul as Biomarkers of Cachexia Induced by AML CIR

**FIGURE 6:**
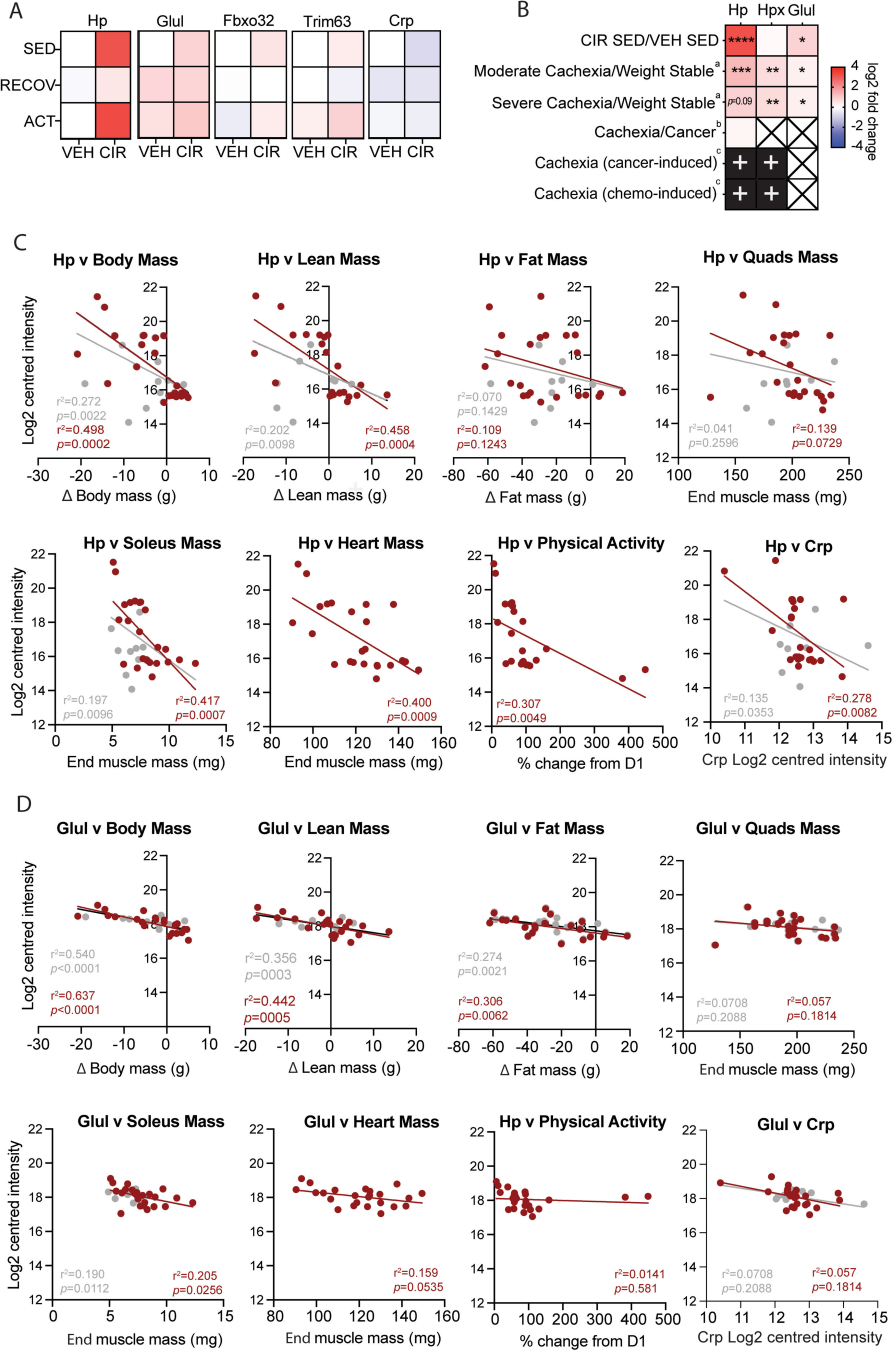
Biomarker potential of muscle haptoglobin (Hp) and glutamine synthetase (Glul) in the context of acute myeloid leukaemia (AML) chemotherapy induction regimen (CIR) cachexia. (A) Conditional heatmap signatures of Hp, Glul, Fbxo32 (Atrogin‐1) and Trim63 (Murf‐1) in AML CIRL and vehicle (VEH) treated muscles. (B) Relative Hp, haemopexin (Hpx) and Glul expression in mined proteomics data sets of rodent and human cancer cachexia. Correlations of (C) Hp and (D) Glul expression with cachexia hallmarks. Grey dots/regression lines denote data points from VEH and CIR groups under both 24 h post‐treatment and 2 weeks recovery conditions, and red data points/regression lines denote VEH and CIR under 24 h post‐treatment conditions only. *p < 0.05, **p < 0.01 and ***p < 0.001 between group differences; heatmap X = protein undetected, + = protein detected but abundance could not be calculated from the data provided. ^a^Proteomics data from C26 tumour cachexia rodent model described in [21], ^b^proteomics data from human cachexia described in [22] and ^c^proteomics data from C26 tumour‐ and chemotherapy‐induced chemotherapy rodent models described in [23].

Hp was the most responsive and labile muscle protein to AML CIR treatment of the > 4700 detected within our proteome (Figure 6A). Hp's scaled heatmap signature for all conditions expressed relative to VEH SED best reflected the scope of cachexia seen across our experimental conditions, where expression was highest under conditions that caused the largest mean reduction in body, lean, fat and muscle mass (Figure 6A). Our data correspond with previously published proteomics data sets of cancer‐ and chemotherapy‐induced cachexia muscle (rodents and humans; Figure 6B), in which both Hp and the analogous haem detoxification protein, haemopexin (Hpx), were increased relative to controls. However, only moderate‐weak correlations were observed between Hp and each of body and lean mass loss from pre‐ to post‐treatment, and there was no correlation with fat mass loss (Figure 6B). Hp better (moderately) correlated with lean (*r* ^2^ = 0.458, *p* = 0.0004; Figure 6C), slow‐twitch soleus (*r* ^2^ = 417, *p* = 0.0007; Figure 6C) and heart (*r* ^2^ = 0.400, *p* = 0.0009; Figure 6C) mass when the recovery readouts were removed from the data set (denoted by red dots and regression line), indicating it may be a better predictor of CIR‐specific molecular pathology and associated muscle mass decrements. Hp only weakly correlated with physical fatigue (*r* ^2^ = 0.307, *p* = 0.0049) and muscle c‐reactive protein (Crp; *r* ^2^ = 0.278, *p* = 0.0082) expression, both hallmarks of cachexia (Figure 6C). There was a weak correlation between Hp expression and endpoint raw quadriceps (matched for the same muscle used for proteomics; Figure 6C), gastrocnemius, EDL and TA mass when recovery data points were removed (Figure 6C and Figure S1). Glul expression was less responsive to treatment than Hp, failing to respond to CIR withdrawal during the recovery period or the added stress of exercise on top of CIR. Glul expression increased in VEH quadriceps over the 2‐week recovery period and due to physical activity, consistent with growth‐ and physical activity‐related metabolism increases. Glul was strongly correlated with body mass (when recovery data were removed, moderately correlated with all data included), moderately correlated with lean mass (*r* ^2^ = 0.442, *p* = 0.0005) and weakly correlated with fat mass change from pre‐ to post‐treatment (*r* ^2^ = 0.306, *p* = 0.0062; Figure 6D) and endpoint mass of the soleus, EDL and plantaris muscles (Figure 6D and Figure S1B), indicating its expression is relative to the overall metabolic state based on body mass. Glul expression did not correlate with endpoint quadriceps (matched proteomics muscle) or heart mass, or with cachexia hallmarks, physical activity and Crp (Figure 6D). CIR‐induced heatmap signatures of atrophy mediators, Fbxo32 (Atrogin‐1) and Trim63 (Murf‐1), mimicked the expression pattern of Hp but not Glul (Figure 6A), suggesting Hp sensitively recapitulates atrophy signalling. Exercise had no effect on Hp nor Glul expression (Figure 5F and Figure S2A,B).

## 4 Discussion

Haematological cancers, such as AML, are rarely studied in the context of cachexia due to the rapid turnaround from diagnosis to initiation of chemotherapy treatment and evaluation for HCT, and our study attempted to tackle this aspect. Despite only lasting 1 week, we reveal that the AML CIR induces a remarkable level of cachexia involving the definitive hallmarks [1]. We show an acute reduction of body, lean and fat mass underpinned by skeletal muscle atrophy, hypermetabolism and muscle and fat catabolism. Mice appear to self‐execute a tissue preservation mechanism by lowering physical activity and energy expenditure to reduce the catabolic insult akin to the human ‘sickness response’ [3]. Persistent hypercatabolism of fat and muscle despite reduced activity and energy expenditure was a curious finding in our study, but mechanistic clues emerged from our proteomics screen. From a fat mass perspective, Acad 11 and 10—recently linked to coordinated lipid peroxidation‐mediated 4‐HA metabolism between peroxisomes and mitochondria [15]—were upregulated in our β‐oxidation pathway analysis (Acad11 especially so). Lipid peroxidation is a well‐established outcome of anthracycline‐mediated reactive oxygen species production [17], and 4‐HA species may be a by‐product, although it is unclear whether increased 4‐HA metabolism is a defensive mechanism that spares muscle from lipotoxic myopathy, is purposeful to fuel thermogenic mitochondrial uncoupling, or both. These enzymes, along with thermogenic proteins (e.g., Ucp1, Sln, Ryr1 and Atp2A1) may be targetable to mitigate cachexia‐related fat loss.

At the muscle level, upregulation of Glul, which enzymatically removes ammonia from the muscle purine nucleotide cycle during conversion of glutamate to glutamine, was a distinct outcome of CIR treatment. Glutamine release from skeletal muscle escalates during proteolysis [24], but cytarabine metabolism also generates significant ammonia via deamination [25] that may necessitate more Glul as an adaptive stress response. As muscle mass declines, the cytarabine‐generated ammonia load may overwhelm adaptive Glul overexpression and either drive or be symptomatic of muscle wasting. Ammonia accumulation is linked to multiple muscle wasting conditions [26]. Additionally, anthracyclines are metabolised by mit‐CI, and we speculated that subunit proteins might be upregulated to effectively process the daunorubicin load. NADH‐ubiquinone oxidoreductase subunits, especially subunit V2 (Ndufv2), were upregulated as previously shown in the context of doxorubicin treatment to protect against cardiomyopathy [18]. We also showed upregulation of Ucp 1 and 3, which drive heat over energy (and CO_2_) production explaining why catabolism increased while energy expenditure (measured via respirometry) reduced. It is difficult to differentiate whether mitochondrial uncoupling is fundamentally important to limit oxidative stress linked to daunorubicin metabolism by mit‐CI or a thermogenic mechanism is activated as fat mass reduces and physical activity levels decline. In the latter scenario, mitochondrial uncoupling likely offsets an increased propensity for hypothermia. Our data suggest that a consequence of these chemotherapy‐induced adaptations is inadvertent systemic hypermetabolic muscle catabolism. Defending the skeletal muscle BCAA pool from chemotherapy‐induced ubiquitylation of L‐type amino acid transporter 1 (LAT1) is a promising approach recently explored in vitro [27] and may be useful in the context of the AML ‘7 + 3’ CIR. This mechanism may link back to upregulated Glul, whereby a futile increase in skeletal muscle glutamine exchange for BCAAs is facilitated by LAT1.

Through unbiased proteomic profiling of quadriceps muscle, we reveal two potential novel biomarkers of AML CIR‐induced cachexia. Hp positively correlates with loss of body, lean and muscle mass and is highly responsive to conditional induction, recovery and exacerbation of AML CIR‐mediated cachexia. Under normal physiological conditions, Hp is synthesised by the liver, lungs and kidneys in response to erythrocyte degradation and circulated in the plasma as an acute‐phase protein to detoxify free haemoglobin alongside its analogue, Hpx [28]. Hp's role in skeletal muscle is poorly understood, although Hpx is a known atrophy myokine [29]. Data from Zip14 metal transporter ablated mice suggest that muscle level Hp expression is sensitive to inflammation‐induced stress response signalling [30], and our data confirm that Hp expression is highest when heat‐shock proteins are also upregulated, that is, with voluntary wheel running, although we saw no upregulation of inflammatory cytokines or Crp in our muscle. Muscle‐specific transcription of Hp has been proven in C26 colon, BAF3 lung, and KP53 pancreatic cancer models [31] and following Zip14 ablation [30]. In the context of cancer cachexia, which in humans frequently involves cancer and chemotherapy elements, muscle Hp protein is detectable in cachexic patients but not significantly more so than noncachexic patients (summarised in Figure 6B) [22]. Notably, this study surveyed only *n* = 4 cachexic cancer patients with unspecified treatment exposure. In sarcopenic congestive heart failure patients, serum Hp 1–1 phenotype significantly correlates with appendicular skeletal muscle index (ASMI) but not hand grip strength, while Hp 2–1 and −2 phenotype negatively associates with ASMI and hand grip strength [32], indicating potential prognostic value of the Hp2 isoform for cachexia. In mice, muscle Hp expression is increased in moderate and severe cachexia induced by C26‐colon carcinoma (summarised in Figure 6B) [21, 23] and specifically within the myofibrillar fraction (in healthy mice, Hp is detected only in the sarcoplasmic fraction) of the myofibre [31]. Corresponding with our data from AML CIR muscle, Hp also increases in response to FOLFIRI (folinic acid (leucovorin) + 5‐fluorouracil+irinotecan) treatment [23]. It is unclear whether there are additive effects of cancer and chemotherapy on muscle‐specific Hp expression since no study has investigated this aspect. Interestingly, Hp abundance is unaffected by Atrogin‐1‐ and Murf‐1‐dependent immobilisation‐atrophy suggesting that its transcription is invoked by a specific insult to skeletal muscle homeostasis [33]. Oxidative stress‐associated protein carbonylation is apparent in Hp^−/−^ KO mice with muscle atrophy, weakness and fatigue, indicating Hp is redox sensitive [34]. Our data highlight that muscle‐specific Hp expression patterns mimic mitochondrial stress levels and iron accumulation, a hallmark of doxorubicin‐induced cardiotoxicity [35], that is, our pathway analysis revealed upregulated iron sulphur cluster biogenesis. That Hp levels were reducing in some mice by the end of the post‐CIR recovery period indicates its sensitivity to CIR effects on muscle. In contrast, Glul was positively correlated with body wasting but was unresponsive to conditional recovery or exacerbation of CIR‐induced cachexia by exercise. It appears to biomark overall metabolism—for example, it is dramatically upregulated in the tumour microenvironment [36]—but not CIR‐induced hypercatabolism specifically, since it also increased with age‐related body mass accretion and with voluntary exercise training in our VEH mice. Further evaluation of these proteins as predictive and/or prognostic biomarkers of cachexia involving muscle wasting is warranted. While a useful biomarker would typically detect in biofluids (i.e., blood and urine), there is opportunity for muscle‐specific biomarkers to be clinically useful across cancer treatment since AML treatment involves minor surgical procedures that enable access to muscle. There is evidence that muscle Hp and Hpx can be secreted into the bloodstream/extracellular media [29, 37], which would make it a particularly useful biofluid marker of cachexia as recently proposed for Duchenne muscular dystrophy [38]. There is no evidence that Glul is secreted from muscle; however, plasma glutamate/glutamine ratios may predict muscle Glul activity and the cachexia hypercatabolic state.

It was surprising that exercise exacerbated cachexia by driving fat mass loss without beneficial recovery of lean tissue in our CIR‐treated mice. We saw shifts in muscle mass in fast‐twitch but not slow‐twitch muscles with CIR treatment, which directly opposed exercise effects observed in VEH mice, indicating that mitochondria‐dense slow twitch fibres may be predominantly affected by CIR combined with the specific muscle activation pattern used in wheel running. Exercise did, however, reduce Ucp 1 and 3 expression, suggesting that over the longer term, it may be useful to abate hypermetabolism if mitochondrial uncoupling is the primary cause. Exercise is generally positively associated with muscle mass, function and metabolism and is being explored as a strategic approach to prevent cachexia [39]. Our data indicate the modality and potentially the intensity of exercise selected for AML patients while they are being actively treated is an important consideration. Previously, Wehrle et al. compared endurance and resistance training application during chemotherapy induction in 22 (of 29; 24% dropout) acute leukaemia patients and demonstrated that only resistance training improved knee extension strength, whereas endurance and no training reduced it [40]. The study did not measure body composition nor report on whether any participants were, or became, cachexic. In breast cancer patients undergoing chemotherapy treatment, 12 weeks of aerobic exercise (walking) reduced body and fat mass like our study, although notably, cachexia is not so much a problem in this malignancy or with its treatment [41].

## 5 Conclusions

Importantly, we demonstrate the cachexic phenotype of an AML CIR cachexia mouse model illuminating hypermetabolism of BCAAs and fat as drivers through metabolic rewiring and mitochondrial uncoupling for both thermogenesis and antioxidation. We identified Hp especially as a potential muscle‐specific biomarker that could be developed in prognostic tools to monitor the efficacy of interventional therapeutics against cachexia. Validation studies are necessary to map Hp (and Glul) expression against cachexia and recovery time course. Our 2‐week recovery period was insufficient in this regard. Future studies characterising the CIR‐induced phenotype would benefit from (1) body mass recovery–directed endpoints, (2) investigation of multiple CIR cycle impacts as is often used clinically and (3) daily muscle and blood sampling across the AML CIR to tease out the contributions of each of daunorubicin and cytarabine to muscle and plasma proteome changes and determine whether Hp has advantages over traditional serum cachexia biomarkers (e.g., Crp and inflammatory cytokines). Our data also illuminate potential therapeutic avenues against hypermetabolism in the metabolic pathways surrounding Hp (iron metabolism), Glul (urea processing and glutamate/glutamine metabolism), Acad 10 and 11 (4‐HA metabolism) and mitochondrial uncoupling for follow‐up.

## Ethics Statement

Animal studies were approved by the Victoria University Animal Ethics Committee (AEETH17/017) and conformed to Australian standards.

## Conflicts of Interest

E.R. has received consultancy fees from Santhera Pharmaceutical, Epirium Bio and Cure ADSSL1 outside of this work. The other authors declare no conflicts of interest.

## Supporting information


**Figure S1.** Correlations of muscle haptoglobin (Hp) and glutamine synthetase (Glul) expression with endpoint mass of hindlimb muscles. Associations of (A) Hp and (B) Glul expression with gastrocnemius, extensor digitorum longus (EDL), plantaris and tibialis anterior (TA) muscle mass at the conclusion of CIR (red dots/regression line) and following 2 weeks recovery after CIR (grey dots/regression line).
**Figure S2.** Effect of exercise on muscle protein expression. (A) Volcano plot demonstrating the response of the quadriceps muscle proteome to voluntary exercise (wheel running) in the CIR‐treated group. (B) Response of muscle haptoglobin (Hp) and glutamine synthetase (Glul) to voluntary exercise in individual animals.
**Table S1.** Muscle and organ mass/body mass (milligrams per gram) ratios. Data are mean ± SEM.
**Table S2.** All significantly up‐ and downregulated Reactome pathways.

## References

[1] K. Fearon , F. Strasser , S. D. Anker , et al., “Definition and Classification of Cancer Cachexia: An International Consensus,” Lancet Oncology 12 (2011): 489–495. 21296615 10.1016/S1470-2045(10)70218-7

[2] D. G. Campelj , C. A. Goodman , and E. Rybalka , “Chemotherapy‐Induced Myopathy: The Dark Side of the Cachexia Sphere,” Cancers (Basel) 13 (2021): 3615. 34298829 10.3390/cancers13143615 PMC8304349

[3] D. G. Campelj , C. A. Timpani , and E. Rybalka , “Cachectic Muscle Wasting in Acute Myeloid Leukaemia: A Sleeping Giant With Dire Clinical Consequences,” Journal of Cachexia, Sarcopenia and Muscle 13 (2022): 42–54. 34879436 10.1002/jcsm.12880 PMC8818658

[4] N. J. Short , M. E. Rytting , and J. E. Cortes , “Acute Myeloid Leukaemia,” Lancet 392 (2018): 593–606. 30078459 10.1016/S0140-6736(18)31041-9 PMC10230947

[5] K. P. Loh , R. F. Dunne , J. W. Friedberg , and S. G. Mohile , “Integrating Assessment of Sarcopenia Into Decision‐Making for Allogeneic Hematopoietic Cell Transplantation: Ready for Prime Time?,” Journal of the National Cancer Institute 111 (2019): 757–759. 31220297 10.1093/jnci/djy233 PMC6695298

[6] C. G. Goodenough , R. E. Partin , and K. K. Ness , “Skeletal Muscle and Childhood Cancer: Where Are We Now and Where We Go From Here,” Aging Cancer 2 (2021): 13–35. 34541550 10.1002/aac2.12027 PMC8445321

[7] A. E. Hiensch , K. A. Bolam , S. Mijwel , et al., “Doxorubicin‐Induced Skeletal Muscle Atrophy: Elucidating the Underlying Molecular Pathways,” Acta Physiologica (Oxford, England) 229 (2020): e13400. 31600860 10.1111/apha.13400 PMC7317437

[8] R. J. Maral and M. Jouanne , “Toxicology of Daunorubicin in Animals and Man,” Cancer Treatment Reports 65, no. Suppl 4 (1981): 9–18. 6809317

[9] R. R. Ellison , J. F. Holland , M. Weil , et al., “Arabinosyl Cytosine: A Useful Agent in the Treatment of Acute Leukemia in Adults,” Blood 32 (1968): 507–523. 4879053

[10] A. B. Nair and S. Jacob , “A Simple Practice Guide for Dose Conversion Between Animals and Human,” Journal of Basic and Clinical Pharmacy 7 (2016): 27–31. 27057123 10.4103/0976-0105.177703 PMC4804402

[11] D. G. Campelj , C. A. Timpani , A. C. Petersen , A. Hayes , C. A. Goodman , and E. Rybalka , “The Paradoxical Effect of PARP Inhibitor BGP‐15 on Irinotecan‐Induced Cachexia and Skeletal Muscle Dysfunction,” Cancers (Basel) 12, no. 12 (2020): 3810, 10.3390/cancers12123810. 33348673 PMC7766767

[12] C. A. Timpani , D. Debrincat , S. Kourakis , et al., “Loss of Endogenous Estrogen Alters Mitochondrial Metabolism and Muscle Clock‐Related Protein Rbm20 in Female mdx Mice,” FASEB Journal 38 (2024): e23718. 38847487 10.1096/fj.202400329R

[13] D. K. Schweppe , J. K. Eng , Q. Yu , et al., “Full‐Featured, Real‐Time Database Searching Platform Enables Fast and Accurate Multiplexed Quantitative Proteomics,” Journal of Proteome Research 19 (2020): 2026–2034. 32126768 10.1021/acs.jproteome.9b00860 PMC7295121

[14] Y. Perez‐Riverol , C. Bandla , D. J. Kundu , et al., “The PRIDE Database at 20 Years: 2025 Update,” Nucleic Acids Research 53, no. D1 (2025): D543–D553, 10.1093/nar/gkae1011. 39494541 PMC11701690

[15] E. H. Rashan , A. K. Bartlett , D. B. Khana , et al., ACAD10 and ACAD11 Enable Mammalian 4‐Hydroxy Acid Lipid Catabolism. bioRxiv. 2024, 10.1101/2024.01.09.574893. PMC12440821 40537578

[16] H. Porumb and I. Petrescu , “Interaction With Mitochondria of the Anthracycline Cytostatics Adriamycin and Daunomycin,” Progress in Biophysics and Molecular Biology 48 (1986): 103–125. 3029807 10.1016/0079-6107(86)90002-7

[17] J. C. Sorensen , B. D. Cheregi , C. A. Timpani , K. Nurgali , A. Hayes , and E. Rybalka , “Mitochondria: Inadvertent Targets in Chemotherapy‐Induced Skeletal Muscle Toxicity and Wasting?,” Cancer Chemotherapy and Pharmacology 78 (2016): 673–683. 27167634 10.1007/s00280-016-3045-3

[18] M. Yang , M. Abudureyimu , X. Wang , Y. Zhou , Y. Zhang , and J. Ren , “PHB2 Ameliorates Doxorubicin‐Induced Cardiomyopathy Through Interaction With NDUFV2 and Restoration of Mitochondrial Complex I Function,” Redox Biology 65 (2023): 102812. 37451140 10.1016/j.redox.2023.102812 PMC10366351

[19] N. C. Bal , S. Singh , F. C. G. Reis , et al., “Both Brown Adipose Tissue and Skeletal Muscle Thermogenesis Processes Are Activated During Mild to Severe Cold Adaptation in Mice,” Journal of Biological Chemistry 292 (2017): 16616–16625. 28794154 10.1074/jbc.M117.790451 PMC5633124

[20] E. Masiero , L. Agatea , C. Mammucari , et al., “Autophagy Is Required to Maintain Muscle Mass,” Cell Metabolism 10 (2009): 507–515. 19945408 10.1016/j.cmet.2009.10.008

[21] A. V. Khamoui , D. Tokmina‐Roszyk , R. G. Feresin , G. B. Fields , and N. P. Visavadiya , “Skeletal Muscle Proteome Expression Differentiates Severity of Cancer Cachexia in Mice and Identifies Loss of Fragile X Mental Retardation Syndrome‐Related Protein 1,” Proteomics 22 (2022): 2100157. 10.1002/pmic.202100157 35289490

[22] H. A. Ebhardt , S. Degen , V. Tadini , et al., “Comprehensive Proteome Analysis of Human Skeletal Muscle in Cachexia and Sarcopenia: A Pilot Study,” Journal of Cachexia, Sarcopenia and Muscle 8 (2017): 567–582. 28296247 10.1002/jcsm.12188 PMC5566647

[23] R. Barreto , G. Mandili , F. A. Witzmann , F. Novelli , T. A. Zimmers , and A. Bonetto , “Cancer and Chemotherapy Contribute to Muscle Loss by Activating Common Signaling Pathways,” Frontiers in Physiology 7 (2016): 472, 10.3389/fphys.2016.00472. 27807421 PMC5070123

[24] T. W. Chang and A. L. Goldberg , “The Metabolic Fates of Amino Acids and the Formation of Glutamine in Skeletal Muscle,” Journal of Biological Chemistry 253 (1978): 3685–3693. 649596

[25] M. R. Park , H. J. Lee , H. M. Jang , et al., “Cytarabine Induces cachexia With Lipid Malabsorption via Zippering the Junctions of Lacteal in Murine Small Intestine,” Journal of Lipid Research 64 (2023): 100387. 37201659 10.1016/j.jlr.2023.100387 PMC10323926

[26] R. A. Stern , S. Dasarathy , and P. E. Mozdziak , “Ammonia Induces a Myostatin‐Mediated Atrophy in Mammalian Myotubes, but Induces Hypertrophy in Avian Myotubes,” Frontiers in Sustainable Food Systems 3 (2019): 115, 10.3389/fsufs.2019.00115.

[27] S. Mora and O. A. J. Adegoke , “Maintenance of the Branched‐Chain Amino Acid Transporter LAT1 Counteracts Myotube Atrophy Following Chemotherapy,” American Journal of Physiology. Cell Physiology 326, no. 3 (2024): C866–C879, 10.1152/ajpcell.00537.2023. 38284122

[28] D. J. Schaer , F. Vinchi , G. Ingoglia , E. Tolosano , and P. W. Buehler , “Haptoglobin, Hemopexin, and Related Defense Pathways—Basic Science, Clinical Perspectives, and Drug Development,” Frontiers in Physiology 5 (2014): 415, 10.3389/fphys.2014.00415. 25389409 PMC4211382

[29] T. Iki and C. Tohda , “Skeletal Muscle Atrophy Induces Memory Dysfunction via Hemopexin Action in Healthy Young Mice,” Biochemical and Biophysical Research Communications 733 (2024): 150606. 39208645 10.1016/j.bbrc.2024.150606

[30] J. Kim , T. B. Aydemir , F. R. Jimenez‐Rondan , C. H. Ruggiero , M. H. Kim , and R. J. Cousins , “Deletion of Metal Transporter Zip14 (Slc39a14) Produces Skeletal Muscle Wasting, Endotoxemia, Mef2c Activation and Induction of miR‐675 and Hspb7,” Scientific Reports 10 (2020): 4050. 32132660 10.1038/s41598-020-61059-2 PMC7055249

[31] I. S. Massart , G. Paulissen , A. Loumaye , et al., “Marked Increased Production of Acute Phase Reactants by Skeletal Muscle During Cancer Cachexia,” Cancers 12 (2020): 3221. 33142864 10.3390/cancers12113221 PMC7693727

[32] A. Karim , T. Muhammad , I. Shah , J. Khan , and R. Qaisar , “Relationship of Haptoglobin Phenotypes With Sarcopaenia in Patients With Congestive Heart Failure,” Heart, Lung & Circulation 31 (2022): 822–831. 10.1016/j.hlc.2022.01.003 35181229

[33] K. H. Lin , G. M. Wilson , R. Blanco , et al., “A Deep Analysis of the Proteomic and Phosphoproteomic Alterations That Occur in Skeletal Muscle After the Onset of Immobilization,” Journal of Physiology 599 (2021): 2887–2906. 33873245 10.1113/JP281071 PMC8353513

[34] E. Bertaggia , G. Scabia , S. Dalise , et al., “Haptoglobin Is Required to Prevent Oxidative Stress and Muscle Atrophy,” PLoS ONE 9 (2014): e100745. 24959824 10.1371/journal.pone.0100745 PMC4069100

[35] Y. Ichikawa , M. Ghanefar , M. Bayeva , et al., “Cardiotoxicity of Doxorubicin Is Mediated Through Mitochondrial Iron Accumulation,” Journal of Clinical Investigation 124 (2014): 617–630. 24382354 10.1172/JCI72931 PMC3904631

[36] G. W. Kim , D. H. Lee , Y. H. Jeon , et al., “Glutamine Synthetase as a Therapeutic Target for Cancer Treatment,” International Journal of Molecular Sciences 22 (2021): 1701, 10.3390/ijms22041701. 33567690 PMC7915753

[37] M. K. Montgomery , W. De Nardo , and M. J. Watt , “Exercise Training Induces Depot‐Specific Remodeling of Protein Secretion in Skeletal Muscle and Adipose Tissue of Obese Male Mice,” American Journal of Physiology. Endocrinology and Metabolism 325 (2023): E227–E238. 37493472 10.1152/ajpendo.00178.2023

[38] S. Murphy , P. Dowling , M. Zweyer , et al., “Proteomic Profiling of mdx‐4cv Serum Reveals Highly Elevated Levels of the Inflammation‐Induced Plasma Marker Haptoglobin in Muscular Dystrophy,” International Journal of Molecular Medicine 39 (2017): 1357–1370. 28440464 10.3892/ijmm.2017.2952 PMC5428965

[39] S. Tsitkanou , K. A. Murach , T. A. Washington , and N. P. Greene , “Exercise Counteracts the Deleterious Effects of Cancer Cachexia,” Cancers (Basel) 14 (2022): 2512. 35626116 10.3390/cancers14102512 PMC9139714

[40] A. Wehrle , S. Kneis , H. H. Dickhuth , A. Gollhofer , and H. Bertz , “Endurance and Resistance Training in Patients With Acute Leukemia Undergoing Induction Chemotherapy‐A Randomized Pilot Study,” Supportive Care in Cancer 27 (2019): 1071–1079. 30121789 10.1007/s00520-018-4396-6

[41] J. J. Kim , Y. A. Shin , and M. H. Suk , “Effect of a 12‐Week Walking Exercise Program on Body Composition and Immune Cell Count in Patients With Breast Cancer Who Are Undergoing Chemotherapy,” Journal of Exercise Nutrition and Biochemistry 19 (2015): 255–262. 26525495 10.5717/jenb.2015.15092812 PMC4624127

