# Supplementary material for: Haptoglobin and Glutamine Synthetase May Biomark Cachexia Induced by Antiacute Myeloid Leukaemia Chemotherapy
Source: J Cachexia Sarcopenia Muscle. 2025 Jun 5;16(3):e13849. doi: 10.1002/jcsm.13849 (PMC12138270; doi:10.1002/jcsm.13849)
Supplement: Supplementary file 1 — Figure S1. Correlations of muscle haptoglobin (Hp) and glutamine synthetase (Glul) expression with endpoint mass of hindlimb muscles. Associations of (A) Hp and (B) Glul expression with gastrocnemius, extensor digitorum longus (EDL), plantaris and tibialis anterior (TA) muscle mass at the conclusion of CIR (red dots/regression line) and following 2 weeks recovery after CIR (grey dots/regression line). Figure S2. Effect of exercise on muscle protein expression. (A) Volcano plot demonstrating the response of the quadriceps muscle proteome to voluntary exercise (wheel running) in the CIR‐treated group. (B) Response of muscle haptoglobin (Hp) and glutamine synthetase (Glul) to voluntary exercise in individual animals. Table S1. Muscle and organ mass/body mass (milligrams per gram) ratios. Data are mean ± SEM. Table S2. All significantly up‐ and downregulated Reactome pathways. [file JCSM-16-e13849-s001.pdf]

Haptoglobin and glutamine synthetase may biomark cachexia induced by anti-acute myeloid leukemia chemotherapy

Dean G. Campelj[1,2,3], Cara A. Timpani[1,4,5], Guinevere Spiesberger[1,4], Luke E. Formosa[6], Joel R. Steele[7], Haijian Zhang[7], Ralf B. Schittenhelm[7], Lewis Leow[4,5], Craig A. Goodman[8], Emma Rybalka[1,4,5]*

[1]Institute for Health and Sport, Victoria University, Melbourne, Victoria, 8001, Australia

[2]Biology of Ageing Laboratory, Centre for Healthy Ageing, Centenary Institute, Camperdown, New South Wales, 2050, Australia

[3]Faculty of Medicine and Health, Charles Perkins Centre, University of Sydney, Sydney, 2050, Australia

[4]Inherited and Acquired Myopathies Program, Australian Institute for Musculoskeletal Science, St Albans, Victoria, 3021, Australia

[5]Department of Medicine—Western Health, Melbourne Medical School, The University of Melbourne, St Albans, Victoria, 3021, Australia

[6]Department of Biochemistry and Molecular Biology, Monash Biomedicine Discovery Institute, Monash University, Clayton, Victoria, 3168, Australia

[7]Monash Proteomics and Metabolomics Platform, Department of Biochemistry and Molecular Biology, Monash University, Clayton, Victoria, 3168, Australia

[8]Centre for Muscle Research, and Department of Anatomy and Physiology, The University of Melbourne, Parkville, Victoria, 3010, Australia

*Corresponding author: emma.rybalka@vu.edu.au, +61 3 83958226

Supplemental figures:

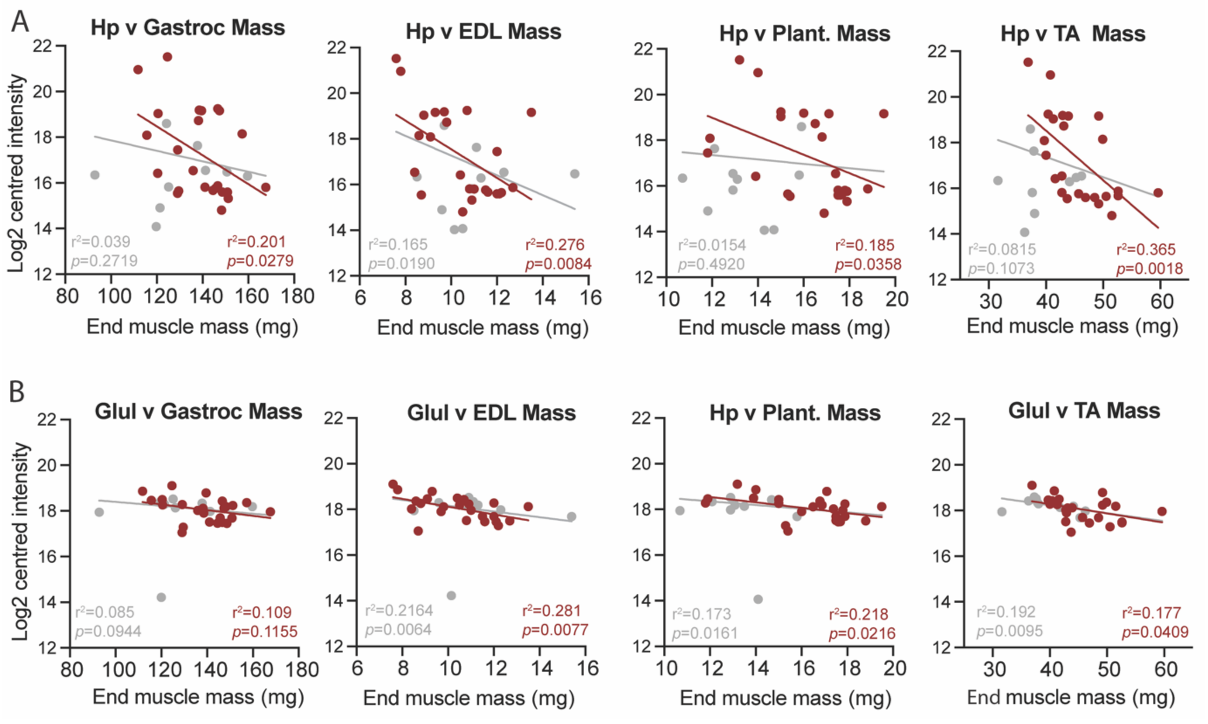

Supplemental Figure 1. Correlations of muscle haptoglobin (Hp) and glutamine synthetase (Glul) expression with endpoint mass of hindlimb muscles. Associations of (A) Hp and (B) Glul expression with gastrocnemius, extensor digitorum longus (EDL), plantaris and tibialis anterior (TA) muscle mass) at the conclusion of CIR (red dots/regression line) and following two weeks recovery after CIR (grey dots/regression line).

A

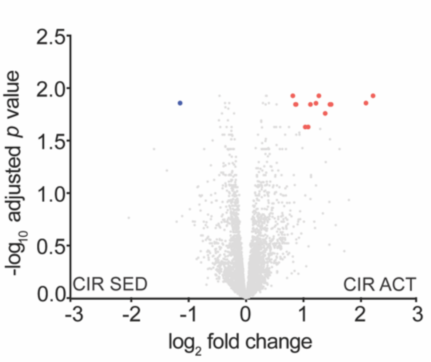

Protein	Log₂ Fold Change	-log₁₀ adjusted *p* value
Hspa1a	2.21	1.543
Csrp3	2.09	1.488
Mustn1	1.48	1.477
Smtnl1	1.46	1.477
Thbs1	1.38	1.408
Myl6b	1.27	1.543
Gusb	1.22	1.486
Hspb1	1.12	1.477
Dnaja4	1.08	1.306
Angptl7	1.03	1.306
Tubb6	0.872	1.477
Lgals3	0.861	1.477
Ablim1	0.814	1.543
Mylk4	-1.15	1.488

B

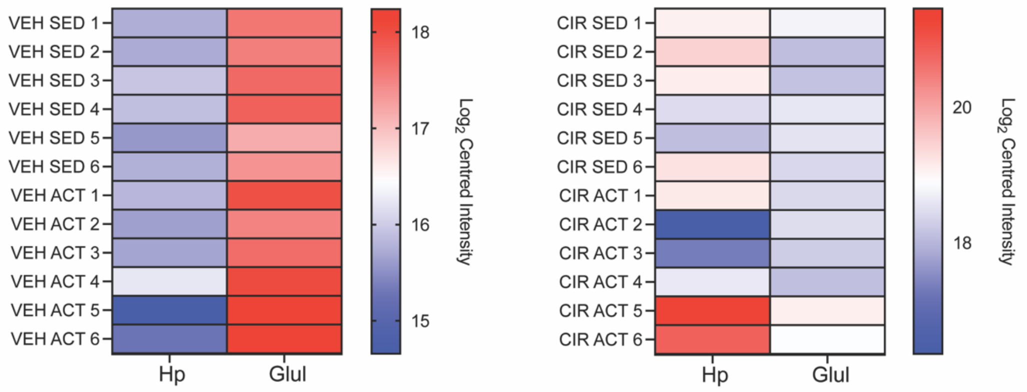

Supplemental Figure 2. Effect of exercise on muscle protein expression. (A) Volcano plot demonstrating the response of the quadriceps muscle proteome to voluntary exercise (wheel running) in the CIR treated group. (B) Response of muscle haptoglobin (Hp) and glutamine synthetase (Glul) to voluntary exercise in individual animals.

Supplemental tables:

Supplemental Table 1: Muscle and organ mass/body mass (mg/g) ratios. Data are mean ± SEM.

	VEH	CIR
Muscles		
Extensor Digitorum Longus (EDL)	0.419 ± 0.007	0.451 ± 0.011
Soleus (SOL)	0.299 ± 0.013	0.307 ±0.010
Tibialis Anteriot (TA)	1.787 ± 0.029	1.903 ± 0.025
Tissues/Organs		
Diaphragm (DIA)	2.834 ± 0.151	3.421 ± 0.185
Heart (HRT)	4.718 ± 0.128	4.742 ± 0.103
Spleen (SPLN)	3.206 ± 0.118	2.421 ± 0.059
Kidney (KID)	7.655 ± 0.149	7.201 ± 0.165
Epidydimal Fat (EPI)	19.340 ± 1.466	18.065 ± 0.624
Liver (LIV)	48.088 ± 2.057	45.781 ± 1.630

Supplemental Table 2: All significantly up- and downregulated Reactome pathways.

Pathway	Direction	Number of Proteins Mapped	FDR
TNFR1-mediated ceramide production	Down	95	1.647E-17
Formation of a pool of free 40S subunits	Down	163	1.314E-12
Eukaryotic Translation Termination	Down	143	1.713E-12
GTP hydrolysis and joining of the 60S ribosomal subunit	Down	188	1.394E-11

Selenocysteine synthesis	Down	140	1.394E-11
L13a-mediated translational silencing of Ceruloplasmin expression	Down	201	1.430E-11
Peptide chain elongation	Down	145	1.501E-11
Eukaryotic Translation Initiation	Down	213	3.495E-11
Cap-dependent Translation Initiation	Down	213	3.495E-11
Nonsense Mediated Decay (NMD) independent of the Exon Junction Complex (EJC)	Down	181	3.495E-11
SRP-dependent cotranslational protein targeting to membrane	Down	168	7.502E-11
Viral mRNA Translation	Down	156	1.456E-10
Eukaryotic Translation Elongation	Down	160	1.487E-10
Response of EIF2AK4 (GCN2) to amino acid deficiency	Down	185	3.864E-10
Selenoamino acid metabolism	Down	183	1.778E-08
Nonsense-Mediated Decay (NMD)	Down	229	2.274E-08
Nonsense Mediated Decay (NMD) enhanced by the Exon Junction Complex (EJC)	Down	229	2.274E-08
Formation of the ternary complex, and subsequently, the 43S complex	Down	113	1.360E-07
Regulation of expression of SLITs and ROBOs	Down	344	1.370E-07
Major pathway of rRNA processing in the nucleolus and cytosol	Down	246	1.425E-07
rRNA processing in the nucleus and cytosol	Down	254	3.020E-07

Translation initiation complex formation	Down	145	7.466E-07
Ribosomal scanning and start codon recognition	Down	130	1.279E-06
Activation of the mRNA upon binding of the cap-binding complex and eIFs, and subsequent binding to 43S	Down	148	1.518E-06
rRNA processing	Down	276	5.877E-06
Cellular response to starvation	Down	243	1.191E-05
Branched-chain amino acid catabolism	Up	36	4.275E-05
SARS-CoV-1 modulates host translation machinery	Down	110	6.946E-05
Signalling by ROBO receptors	Down	526	1.615E-04
Nuclear Receptor transcription pathway	Down	251	3.248E-04
Transcription of SARS-CoV-2 sgRNAs	Down	107	4.676E-04
Influenza Viral RNA Transcription and Replication	Down	282	5.100E-04
RHO GTPases regulate CFTR trafficking	Down	533	5.555E-04
RUNX1 regulates transcription of genes involved in WNT signalling	Down	191	5.635E-04
Defective CFTR causes cystic fibrosis	Down	676	9.779E-04
RUNX1 regulates estrogen receptor mediated transcription	Down	198	1.018E-03
SARS-CoV-2 modulates host translation machinery	Down	104	1.124E-03
Regulation of RUNX2 expression and activity	Down	409	1.187E-03
Cross-presentation of soluble exogenous antigens (endosomes)	Down	111	1.315E-03
NIK-->noncanonical NF-kB signalling	Down	230	2.342E-03

Regulation of ornithine decarboxylase (ODC)	Down	109	2.342E-03
ABC transporter disorders	Down	699	2.396E-03
Translation	Down	468	2.411E-03
SARS-CoV-2 Genome Replication and Transcription	Down	142	2.525E-03
Dectin-1 mediated noncanonical NF-kB signalling	Down	249	2.889E-03
ABC-family proteins mediated transport	Down	712	3.035E-03
Mitochondrial protein import	Up	156	3.462E-03
Influenza Infection	Down	386	4.032E-03
Autodegradation of the E3 ubiquitin ligase COP1	Down	226	4.659E-03
The citric acid (TCA) cycle and respiratory electron transport	Up	304	4.783E-03
Oxygen-dependent proline hydroxylation of Hypoxia-inducible Factor Alpha	Down	199	4.979E-03
Citric acid cycle (TCA cycle)	Up	35	5.600E-03
Metabolism of polyamines	Down	128	6.683E-03
SUMOylation of intracellular receptors	Down	285	6.683E-03
Stabilization of p53	Down	249	7.596E-03
Late endosomal microautophagy	Down	710	7.664E-03
Nef-mediates down modulation of cell surface receptors by recruiting them to clathrin adapters	Down	69	8.453E-03
G1/S DNA Damage Checkpoints	Down	275	8.925E-03
Ub-specific processing proteases	Down	1018	8.925E-03

TNFR1-induced NF-kappa-B signaling pathway	Down	345	8.925E-03
Transcriptional regulation by RUNX2	Down	595	8.925E-03
Clathrin-mediated endocytosis	Down	959	8.925E-03
Disorders of transmembrane transporters	Down	772	8.925E-03
Cargo recognition for clathrin-mediated endocytosis	Down	902	8.925E-03
Beta-oxidation of pristanoyl-CoA	Up	9	9.366E-03
Cellular response to hypoxia	Down	223	9.366E-03
Vif-mediated degradation of APOBEC3G	Down	172	9.366E-03
Aggrephagy	Down	742	9.423E-03
p53-Dependent G1 DNA Damage Response	Down	271	9.423E-03
p53-Dependent G1/S DNA damage checkpoint	Down	271	9.423E-03
Autodegradation of Cdh1 by Cdh1:APC/C	Down	161	9.423E-03
Ovarian tumor domain proteases	Down	442	9.423E-03
Constitutive Signalling by Aberrant PI3K in Cancer	Down	444	9.423E-03
FBXL7 down-regulates AURKA during mitotic entry and in early mitosis	Down	192	1.012E-02
Mitochondrial Fatty Acid Beta-Oxidation	Up	61	1.012E-02
Regulation of RUNX3 expression and activity	Down	217	1.012E-02
Mitochondrial iron-sulfur cluster biogenesis	Up	39	1.012E-02
TFAP2 (AP-2) family regulates transcription of growth factors and their receptors	Down	350	1.012E-02

Ubiquitin Mediated Degradation of Phosphorylated Cdc25A	Down	160	1.012E-02
p53-Independent DNA Damage Response	Down	160	1.012E-02
p53-Independent G1/S DNA damage checkpoint	Down	160	1.012E-02
mitochondrial fatty acid beta-oxidation of saturated fatty acids	Up	14	1.299E-02
UCH proteinases	Down	293	1.385E-02
APC/C:Cdc20 mediated degradation of Securin	Down	165	1.385E-02
Regulation of TNFR1 signalling	Down	421	1.547E-02
NOD1/2 signalling Pathway	Down	285	1.662E-02
APC/C:Cdh1 mediated degradation of Cdc20 and other APC/C:Cdh1 targeted proteins in late mitosis/early G1	Down	222	1.782E-02
Selective autophagy	Down	879	1.782E-02
TGF-beta receptor signalling activates SMADs	Down	318	1.810E-02
Anchoring fibril formation	Down	31	1.810E-02
Deubiquitination	Down	1227	1.810E-02
Cristae formation	Up	82	1.810E-02
Axon guidance	Down	1070	1.854E-02
Chaperone Mediated Autophagy	Down	769	1.854E-02
CLEC7A (Dectin-1) signalling	Down	358	1.858E-02
SCF-beta-TrCP mediated degradation of Emi1	Down	196	1.902E-02
PI3K/AKT signalling in Cancer	Down	540	1.949E-02

Pyruvate metabolism and Citric Acid (TCA) cycle	Up	99	1.955E-02
Respiratory electron transport, ATP synthesis by chemiosmotic coupling, and heat production by uncoupling proteins.	Up	227	2.024E-02
Nef Mediated CD4 Down-regulation	Down	51	2.061E-02
Crosslinking of collagen fibrils	Down	40	2.061E-02
TNF signalling	Down	457	2.122E-02
Activation of AMPK downstream of NMDARs	Down	95	2.124E-02
CDK-mediated phosphorylation and removal of Cdc6	Down	175	2.136E-02
Nervous system development	Down	1108	2.161E-02
Interleukin-1 signalling	Down	667	2.272E-02
Integrin cell surface interactions	Down	303	2.336E-02
Macroautophagy	Down	938	2.363E-02
Formation of ATP by chemiosmotic coupling	Up	25	2.363E-02
Regulation of activated PAK-2p34 by proteasome mediated degradation	Down	158	2.363E-02
Degradation of DVL	Down	184	2.363E-02
COPI-independent Golgi-to-ER retrograde traffic	Down	89	2.485E-02
Activation of NF-kappaB in B cells	Down	256	2.485E-02
SCF(Skp2)-mediated degradation of p27/p21	Down	221	2.576E-02
RHOQ GTPase cycle	Down	725	2.745E-02
Hh mutants abrogate ligand secretion	Down	182	2.869E-02

mRNA Capping	Down	70	3.175E-02
PI5P, PP2A and IER3 Regulate PI3K/AKT signalling	Down	533	3.213E-02
AUF1 (hnRNP D0) binds and destabilizes mRNA	Down	271	3.280E-02
Nuclear signalling by ERBB4	Down	325	3.313E-02
RHO GTPase Effectors	Down	1077	3.462E-02
Translocation of ZAP-70 to Immunological synapse	Down	22	3.481E-02
Hh mutants are degraded by ERAD	Down	181	3.559E-02
APC:Cdc20 mediated degradation of cell cycle proteins prior to satisfaction of the cell cycle checkpoint	Down	186	3.638E-02
GSK3B and BTRC:CUL1-mediated-degradation of NFE2L2	Down	211	3.756E-02
TNFR2 non-canonical NF-kB pathway	Down	308	3.783E-02
Metabolism of proteins	Down	2358	3.787E-02
Cdc20:Phospho-APC/C mediated degradation of Cyclin A	Down	185	3.960E-02
Degradation of AXIN	Down	176	3.960E-02
Asymmetric localization of PCP proteins	Down	176	4.030E-02
TP53 regulates transcription of additional cell cycle genes whose exact role in the p53 pathway remain uncertain	Down	118	4.047E-02
Hedgehog ligand biogenesis	Down	200	4.048E-02
Orc1 removal from chromatin	Down	211	4.079E-02
Switching of origins to a post-replicative state	Down	230	4.137E-02
Activation of APC/C and APC/C:Cdc20 mediated degradation of mitotic proteins	Down	210	4.137E-02

Negative regulation of the PI3K/AKT network	Down	549	4.137E-02
APC/C:Cdc20 mediated degradation of mitotic proteins	Down	187	4.137E-02
Autophagy	Down	988	4.171E-02
Membrane Trafficking	Down	1582	4.321E-02
Regulation of APC/C activators between G1/S and early anaphase	Down	249	4.389E-02
APC/C-mediated degradation of cell cycle proteins	Down	275	4.444E-02
Regulation of mitotic cell cycle	Down	275	4.444E-02
Transcriptional regulation by RUNX1	Down	625	4.521E-02
Death Receptor signalling	Down	686	4.731E-02
ALK mutants bind TKIs	Down	72	4.731E-02
Somitogenesis	Down	165	4.942E-02
Vesicle-mediated transport	Down	1633	4.989E-02
